# Optimal Operation of the Hybrid Electricity Generation System Using Multiverse Optimization Algorithm

**DOI:** 10.1155/2019/6192980

**Published:** 2019-03-11

**Authors:** Muhammad Sulaiman, Sohail Ahmad, Javed Iqbal, Asfandyar Khan, Rahim Khan

**Affiliations:** ^1^Department of Mathematics, Abdul Wali Khan University Mardan, Mardan, KP, Pakistan; ^2^Department of Computer Science, Abdul Wali Khan University Mardan, Mardan, KP, Pakistan

## Abstract

The ongoing load-shedding and energy crises due to mismanagement of energy produced by different sources in Pakistan and increasing dependency on those sources which produce energy using expensive fuels have contributed to rise in load shedding and price of energy per kilo watt hour. In this paper, we have presented the linear programming model of 95 energy production systems in Pakistan. An improved multiverse optimizer is implemented to generate a dataset of 100000 different solutions, which are suggesting to fulfill the overall demand of energy in the country ranging from 9587 MW to 27208 MW. We found that, if some of the power-generating systems are down due to some technical problems, still we can get our demand by following another solution from the dataset, which is partially utilizing the particular faulty power system. According to different case studies, taken in the present study, based on the reports about the electricity short falls been published in news from time to time, we have presented our solutions, respectively, for each case. It is interesting to note that it is easy to reduce the load shedding in the country, by following the solutions presented in our dataset. Graphical analysis is presented to further elaborate our findings. By comparing our results with state-of-the-art algorithms, it is interesting to note that an improved multiverse optimizer is better in getting solutions with lower power generation costs.

## 1. Introduction

Proper management and finding out optimal solutions to utilize the available power production sources is an important optimization problem in Pakistan, which is the main theme of this paper [1–11]. According to the reports in [12, 13], the demand of the power is increasing exponentially and which is causing an increase in load shedding each year [14]. The cause of this shortage is mismanagement of power production sources.

According to a report published by National Electric Power Regulatory Authority (NEPRA) in 2016 [15], the total capacity of 27240 MW is installed in the country, whereas the demand rises to 22000 MW in summer [16]. These production units can produce enough energy if all are managed optimally. In [17], these production units were normally producing up to 13240 MW with 4760 MW of shortfall. In nomenclature, we have indicated all the 95 installed capacities of electricity production with majority of them depending on thermal sources [15]. In [14], it is reported that the gap between demand and production is expected to increase in the coming years. It is further reported in the literature [14, 18] that urban areas are facing a load shading in summer season as of 10–12 hours, and in rural areas, it is even worse as 16–18 hours a day. Urbanization and rapid increase in population have caused an increase in the industrial zones [19], and it is worth noting that the demand of electricity was 16000 (MW) during summer of 2012 while the short fall was 8500 (MW) [20].

In this study, we have explored a scientific decision-making approach in terms of mathematical programming for providing a strategy to utilize the available production sources of Pakistan to meet the demand of energy in different seasons. However, in [21], a general mechanism was presented in terms of 5 major energy production types as *X*_1_ to *X*_5_. They have fixed the per unit price to estimate the overall mathematical model. We have extended the model and included 95 production sources, which generates electricity using different means (oil, gas, wind, hydropower, and coal). We have implemented per unit price according to the actual price of fuel used to run these sources. The production capacities of each source are given in Table 1 [22]. Our model will help to reduce the shortfall by utilizing the power production sources properly.

In the last few years, evolutionary algorithms (EAs) are implemented to solve different real-world optimization problems. EAs simulate social behavior or natural procedures in order to handle optimization problems with best solution. Among the popular EAs are the Bat algorithm [23, 24], grasshopper optimization algorithm (GOA) [25], and firefly algorithm (FA) [26, 27]. The efficiency of metaheuristics is better as compared to classical optimization techniques in solving optimization problems with iterations and random search behavior. The main idea behind all EAs is the survival of the fittest, which in return increases the fitness of individuals in population. EAs are implemented with different frameworks, which includes population structure and search equations [28]. EAs with different frameworks generate random populations. Then, all solutions are evaluated according to a given objective function. The search space is explored and exploited in two phases. EAs get stuck in local optima due to their random search. Several papers are published in the literature to overcome this problem in EAs [29, 30].

The multiverse optimization algorithm (MVO) is a nature-inspired optimization technique given in [31]. The key idea of MVO is inspired from the theory of multiverses. Since its invention, it is applied to solve several real-world problems. In [31], it is shown that MVO obtained very competitive results compared with other metaheuristic optimization algorithms. Also, Faris et al. [32] employed the MVO for training the multilayer perceptions in neural network. The efficiency of their techniques was evaluated on different medical datasets from UCI repository. The outcome of their experiments is then compared to different metaheuristics, namely, Particle Swarm Optimization (PSO), Genetic Algorithm (GA), Differential Evolution (DE) Algorithm, and Cuckoo Search (CS). It was observed that MVO was improved in terms of convergence and avoidance of local optima. MVO still lacks behind in accuracy of results and convergence speed. Fewer studies, like Levy-flight-based MVO [33] and a quantum version of MVO [34], are recently proposed to further enhance the capabilities of MVO. Experimental results show that they have improved the solutions to some extent.

Currently, as can be seen in the former review, there are limited works on improving the performance of MVO. This work presents a new method of initialization with the MVO algorithm called improved multiverse optimization algorithm (IMVO). The experimental results on mathematical model of economic dispatch problem of electricity generation system in Pakistan and its three case studies show that IMVO is very competitive over FA, Bat algorithm, and GOA.

Furthermore, we have presented solutions to five case studies for proper management and production by all the 95 energy producers in the country. Our model can be implemented to reduce the load shedding and distribute the burden of production on respective power production units.

This work is organized as follows: Section 2 describes the concept of multiverse optimizer and improvements in it. The mathematical model for the problem is described in Section 3. Experimental settings, results, and discussions based on numerical simulations are given in Section 4. Conclusion of our findings is presented in Section 5.

## 2. An Improved Multiverse Optimizer

**Figure 1:**
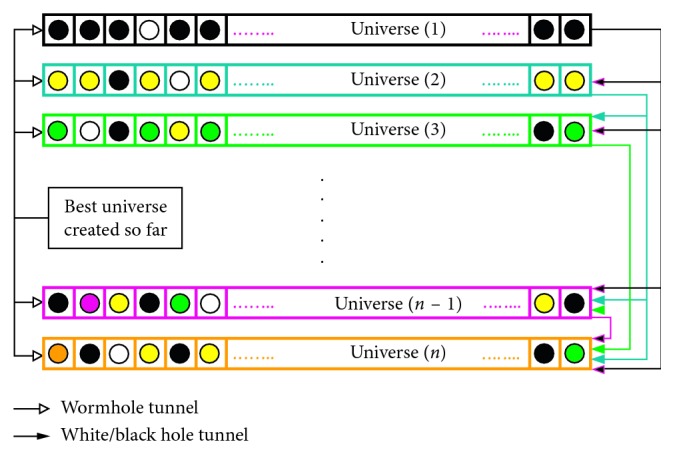
A sketch of MVO [31].

The MVO algorithm simulates the theory of multiverse, where three verses play main part in the whole algorithmic procedure: these are white holes, black holes, and wormholes. This is a population-based algorithm, where the population is searched in two phases: exploration in which the search space is visited very well and exploitation performs the search in neighborhood of a solution extensively. White hole and black hole, in the theory of multiverses, are used as exploration agents, and wormhole is acting as exploitation agent in this algorithm. In Figure 1, black, yellow, green, and pink are the objects which are moving through the white/black hole, and white points represent objects which move through the wormhole. There are 5 assumptions that are applied in MVO to the population/universe [31]:

- The probability of having the white holes is directly proportional to the inflation rate.
- Individuals (universes) having higher inflation rate send objects through white holes with higher probability.
- Individuals (universes) having higher inflation rate send objects through white holes with higher probability.
- Individuals (universes) having lower rate of inflation send objects through black holes with higher probability.
- The objects present in all universes are moved randomly towards the current best individual (universe) through wormholes. This random process occurs without taking into account the inflation rate. A sketch of this algorithm is shown in Figure 1.

The initialization phase of MVO can be written as follows:

(1) $$\begin{array}{c} U_{}=\left[\begin{array}{ccc} x_{1}^{1} & \cdots & x_{1}^{d}\\ \vdots & \ddots & \vdots \\ x_{n}^{1} & \cdots & x_{n}^{d} \end{array}\right], \end{array}$$

where *U* represents the matrix of candidate solutions, *d* is the dimensions of the problem, and *n* is the number of universes (population of solutions) which can be represented as follows:

(2) $$\begin{array}{c} x_{i}^{j}=\left\{\begin{array}{cc} x_{k}^{j}, & \;r1<\text{NI}\left(U_{i}\right)\\ x_{i}^{j}, & \;r1\geq \text{NI}\left(U_{i}\right) \end{array}\right\}, \end{array}$$

where *x*_*i*_^*j*^ is the *j*^th^ parameter of *i*^th^ universe, *U*_*i*_ denotes the *i*^th^ universe, NI (*U*_*i*_) is the scaled inflation rate of the *i*^th^ universe, *r*1 ∈ (01), and *x*_*k*_^*j*^ is the *j*^th^ parameter of *k*^th^ universe selected by a roulette wheel selection mechanism. The objects are changed among the universes without perturbation. To maintain the balance between exploration and exploitation during the searching process in MVO, each universe is randomly treated to have wormholes to transport its objects through space. In order to ensure the exploitation around the current best solutions, particular wormhole tunnels are always established between a universe and the best universe formed so far, which can be represented mathematically as follows:

(3) $$\begin{array}{c} x_{i}^{j}=\left\{\begin{array}{cc} \left\{\begin{array}{cc} X_{J}+\text{TDR}\times \left(\left({\text{ub}}_{j}-{\text{lb}}_{j}\right)\times r_{4}+{\text{lb}}_{j}\right), & \;r_{3}<0.5,\\ X_{J}-\text{TDR}\times \left(\left({\text{ub}}_{j}-{\text{lb}}_{j}\right)\times r_{4}+{\text{lb}}_{j}\right), & \;r_{3}\geq 0.5, \end{array}\right. & \;r_{2}<\text{WEP},\\ x_{i}^{j}, & \;r_{2}\geq \text{WEP}, \end{array}\right. \end{array}$$

where *X*_*j*_ is the *j*^th^ parameter of best universe formed so far, TDR (travelling distance rate) and WEP (wormhole existence probability) are coefficients, lb_*j*_ and ub_*j*_ are the lower bound and upper bound of *j*^th^ variable, respectively, *x*_*ij*_ is the *j*^th^ parameter of *i*^th^ universe and *r*_2_, *r*_3_, and *r*_4_ are random numbers between 0 and 1. WEP and TDR are treated as adaptive formula as follows:

(4) $$\begin{array}{c} \text{WEP}=W_{\min}+l\left(\frac{W_{\max}-W_{\min}}{L}\right),\\ \text{TDR}=1-\frac{l^{1/p}}{L^{1/p}}, \end{array}$$

where *W*_min_ is the minimum (in this paper, it is set to 0.2), *W*_max_ is the maximum (in this paper, it is set to 1), *l* is the current iteration, *L* is the maximum iterations, and *p* is the exploitation accuracy over the iterations (in this paper, it is set to 6). Higher the value of *p*, the sooner and more accurate exploitation/local search is performed [31]. It is worth to highlight that the MVO algorithm depends on number of iterations, number of universes, roulette wheel mechanism, and universe sorting mechanism. The quick sort algorithm is used to sort universe at each iteration, and roulette wheel selection is run for each variable in every universe in all iterations. Detailed description of MVO can be seen in [31].

## 3. Our Proposed Initialization Strategy

According to the trend in stochastic techniques, random initialization in each generation is the frequently used method to initialize algorithms without knowing any priory information about the problem in hand. The idea of random initialization is often pushing the algorithm to explore the given domain very well, while the exploitation around best solutions is not carried out by this type of initialization. We have used an initialization strategy in which IMVO is balanced in terms of exploration and exploitation. We have designed a two-stage initialization strategy: in the first stage, the algorithm is initialized randomly for a certain number of scattered solutions in the whole search domain. In stage two, after the algorithm completes a certain number of iterations and collects best points in the search domain, we initialize the IMVO with population of these best solutions to concentrate the algorithm on exploitation around the best solutions in the space. Hence, in current studies, we have assigned the function evaluations, fixed to solve a problem, in two parts. The first part initializes with random populations and collects the best solutions of each iteration by using the following equation:

(5) $$\begin{array}{c} U_{i}={\text{Lb}}_{i}+\left({\text{Ub}}_{i}-{\text{Lb}}_{i}\right)\;*\text{ rand}\left(0,1\right). \end{array}$$

In the second phase, the algorithm is directed to search in the neighborhood of the best solutions obtained in the first part and exploits the search space around the population of these best solutions.

## 4. Modeling of Electricity Production Cost as a Single Objective Linear Programming Problem

In this paper, we have presented an optimal mixture of energy production system as a linear programming (LP) model consisting of different decision variables, representing different electricity production sources of Pakistan, see Nomenclature [21]. Each variable is bounded and generates electricity through different fuel types like hydro, thermal, nuclear, wind, and solar energies. The suggested LP model includes different production sources as *x*_*i*_,  *i*=1, 2,…, 95 and *u*_*i*_,  *i*=1, 2, 3,…, 95 represent different costs per kWh for each source. The objective function represents the total cost (*C*) incurred to meet the demand of energy in different situations. Mathematically,

(6) $$\begin{array}{c} \text{minimize cost}=\overset{95}{\underset{i=1}{\sum }}u_{i}x_{i}, \end{array}$$

where *u*_*i*_ represents the cost per MWh and *x*_*i*_ are the different sources given in nomenclature. The objective of this study is to produce a data set of solutions so that we can select the required optimal solution fulfilling the given demand with lowest cost per kWh as compared to other solutions in the data set. For more details, about the data set generated, the reader may refer to [35]. The problem is to search for best solutions among several candidate solutions and to meet the required power demand and satisfy constraints on each variable. These constraints are given in Equation (7) and Table 1 as follows.

Total demand constraint:

(7) $$\begin{array}{c} \overset{95}{\underset{t=1}{\sum }}x_{t}\geq d. \end{array}$$

## 5. Experimental Settings, Results, and Discussion

**Figure 2:**
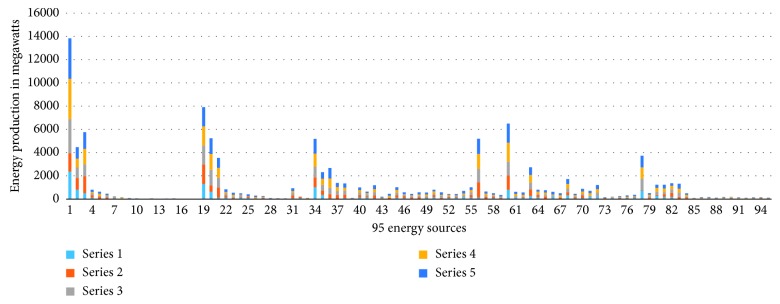
A weighted comparison of the 95 energy sources used in five case studies. Series 1–5 represent case studies 1–5, respectively.

**Figure 3:**
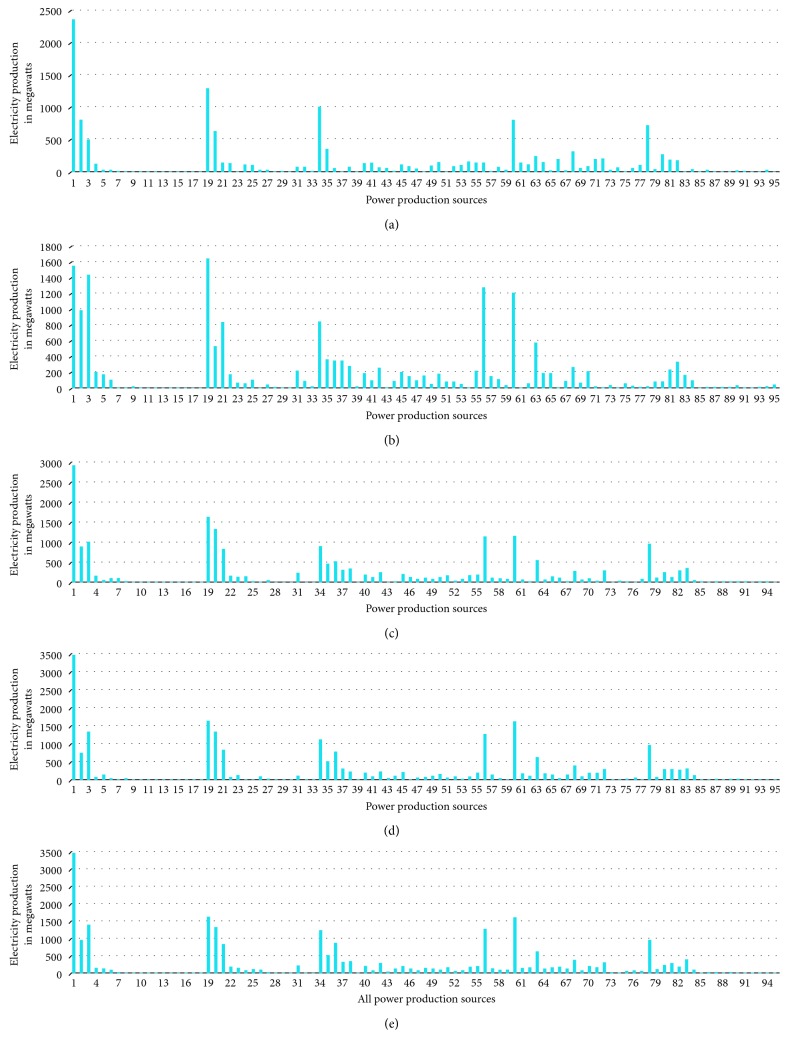
Comprehensive plots of five case studies discussed in Table 4. All production sources are plotted against the electricity production in megawatts. (a) Case Study 1: demand of 15003 MW. (b) Case Study 2: demand of 19003 MW. (c) Case Study 3: demand of 23000 MW. (d) Case Study 4: demand of 25000.3 MW. (e) Case Study 5: demand of 27208.3 MW.

**Figure 4:**
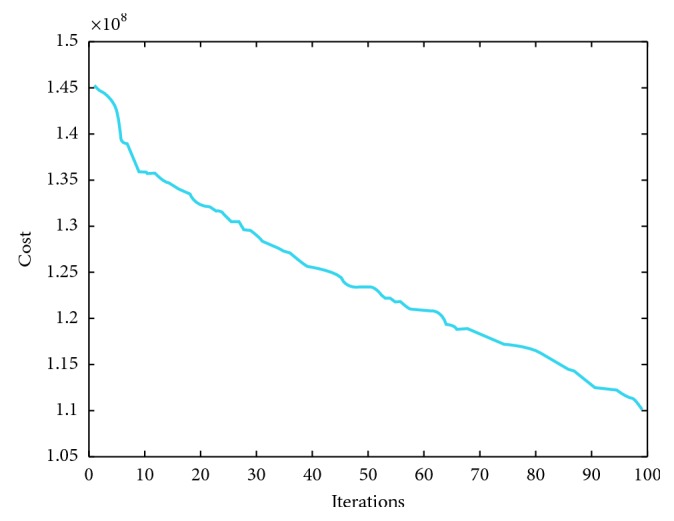
IMVO convergence graph demand for 15000 megawatt.

**Figure 5:**
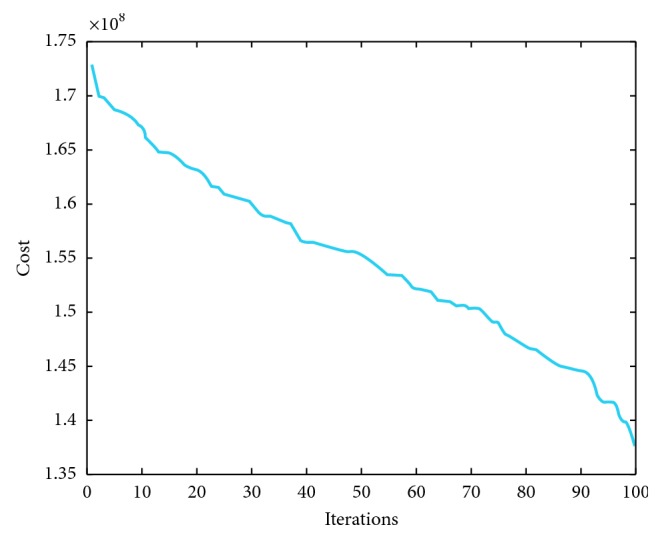
IMVO convergence graph demand for 19000 megawatt.

**Figure 6:**
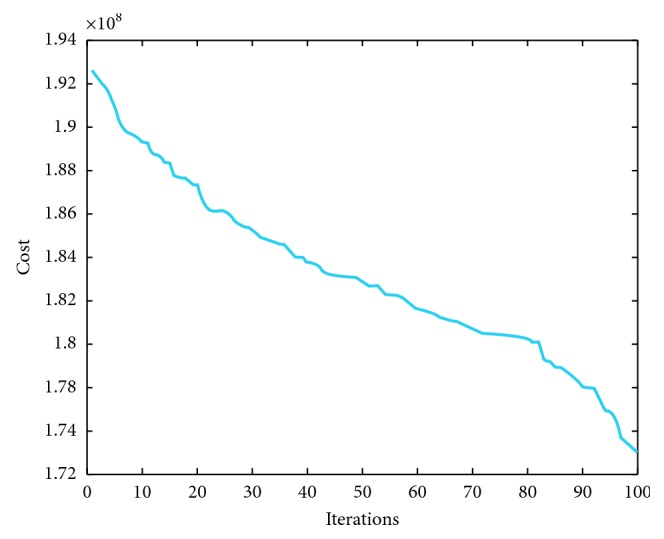
IMVO convergence graph demand for 23000 megawatt.

We have implemented IMVO which is coded in Matlab R2016 [31]. The main features of our computer included 8 GB RAM with Intel® Core™ i7-7500U CPU @ 2.7 GHz 2.9 GHz with a 64-bit operating system Windows 10. For a fair comparison, the population size was taken as 100 and the number of iterations was 100. We have repeated our simulations 10 times. To elaborate the efficiency of IMVO, we have considered three special cases. The experimental outcome is compared with state-of-the-art algorithms: firefly algorithm, bat algorithm, and grasshopper optimization algorithm, as in Tables 2, 3, and 4. Also, we have picked 5 solutions from overall 100000 solutions and presented these solutions as different case studies with their respective costs per MWh (Table 5), which are based on the power demand in summers as provided in [20, 36–38]. The power demands and decision variables, which represent the 95 production sources in Pakistan and cost incurred along with the total power produced, are given in Table 5. The individual variations of 20 best variables required to produce a particular solution in all case studies are furnished in Table 6, and a weighted comparison of the 95 energy sources is given in Figure 2. Looking at these graphs, the reader can deduce the importance of each production source of electricity in national grid of the country. One can deduce which solution to choose if a particular source is down due to some fault or which sources are very impotent and acting as a backbone in generating the required electricity. We have depicted five case studies graphically as shown in Figure 3. These graphs are collective representation of 95 production sources acting to produce a certain demand. It is interesting to note that the production of electricity from available sources is manageable, if proper techniques, like IMVO algorithm, are implemented to solve the problem. It is worth to note that we have pointed out the top 20 production sources of electricity in Pakistan according to the five case studies listed as in Table 5. These sources play vital part in the energy production as shown in Table 6. It is obvious that certain sources like *x*_1_ are producing higher demand which are overburdened and could be relaxed by installing or expanding alternate sources. In Table 7, prices depending on different fuel types are listed for a megawatt of electricity. The convergence plots for these three case studies are depicted in Figures 4–6. It is interesting to note that, in all cases with demands of 15000, 19000, and 23000 megawatts, IMVO produced better solutions with lowest costs in millions (Table 2–4).

## 6. Conclusions

In this paper, an improved multiverse optimization algorithm is implemented for solving a linear programming model for proper management of electricity production sources in Pakistan. To address the proper management of power production sources, several bound constraints, such as constraint for total demand, and bounds on all variables are considered. The objective function is linear with 95 decision variables and different coefficients. The IMVO algorithm is easy to implement with few parameters. We have found solutions for five different case studies and analyzed all solutions graphically. A link to the data set of 100000 different solutions is provided in this paper. In our results, we have obtained better solutions to meet the power demand in summer. It is recommended that current resources can produce the required power demand but some of the resources are required to be expanded in capacity along with installation of new sources. In future, we intend to include other factors, like power loss, in our model.

## Figures and Tables

**Table 1:** Electricity production constraints on each source.

Power unit	Lower limit	Upper limit
*x*_1_	0	3478
*x*_2_	0	1000
*x*_3_	0	1450
*x*_4_	0	243
*x*_5_	0	184
*x*_6_	0	130
*x*_7_	0	121
*x*_8_	0	72
*x*_9_	0	30
*x*_10_	0	22
*x*_11_	0	22
*x*_12_	0	20
*x*_13_	0	17
*x*_14_	0	14
*x*_15_	0	13.5
*x*_16_	0	4
*x*_17_	0	1
*x*_18_	0	1
*x*_19_	0	1655
*x*_20_	0	1350
*x*_21_	0	850
*x*_22_	0	244
*x*_23_	0	195
*x*_24_	0	174
*x*_25_	0	150
*x*_26_	0	132
*x*_27_	0	59
*x*_28_	0	39
*x*_29_	0	35
*x*_30_	0	17
*x*_31_	0	247
*x*_32_	0	100
*x*_33_	0	100
*x*_34_	0	1260
*x*_35_	0	560
*x*_36_	0	900
*x*_37_	0	362
*x*_38_	0	365
*x*_39_	0	29
*x*_40_	0	225
*x*_41_	0	165
*x*_42_	0	330
*x*_43_	0	94
*x*_44_	0	152.5
*x*_45_	0	226.5
*x*_46_	0	157
*x*_47_	0	110
*x*_48_	0	179
*x*_49_	0	165
*x*_50_	0	188
*x*_51_	0	201.5
*x*_52_	0	136
*x*_53_	0	140
*x*_54_	0	225
*x*_55_	0	225
*x*_56_	0	1292
*x*_57_	0	165
*x*_58_	0	120
*x*_59_	0	131
*x*_60_	0	1638
*x*_61_	0	232
*x*_62_	0	202
*x*_63_	0	660
*x*_64_	0	200
*x*_65_	0	200
*x*_66_	0	225
*x*_67_	0	164
*x*_68_	0	412
*x*_69_	0	114
*x*_70_	0	225
*x*_71_	0	235
*x*_72_	0	350
*x*_73_	0	52
*x*_74_	0	80
*x*_75_	0	110
*x*_76_	0	133.5
*x*_77_	0	126
*x*_78_	0	990
*x*_79_	0	137
*x*_80_	0	325
*x*_81_	0	325
*x*_82_	0	340
*x*_83_	0	425
*x*_84_	0	150
*x*_85_	0	50
*x*_86_	0	50
*x*_87_	0	49
*x*_88_	0	50
*x*_89_	0	52
*x*_90_	0	50
*x*_91_	0	56
*x*_92_	0	30
*x*_93_	0	49
*x*_94_	0	50
*x*_95_	0	50

**Table 2:** Case Study A: demand for 15000 megawatt, where *S*_1_ − *S*_8_ represent the collection of sources operating on 8 fuel types.

	Firefly	BAT	GOA	IMVO
Price in PKR	390.831986	252.289853	201.3324084	**111.5048004**
Variable (MW)	15017.4015	15006.4398	15002.26667	15067.46055
*S*_1_	4035.19978	2893.04991	3513.562502	4785.656
*S*_2_	1407.73428	1873.28453	1072.160605	1338.551
*S*_3_	981.013264	158.209497	62.4800805	431.9034
*S*_4_	1137.47918	567.079361	395.4767659	1476.913
*S*_5_	5684.88097	8502.04227	8812.308041	5366.239
*S*_6_	921.438836	523.099019	684.1945259	1180.03
*S*_7_	393.294457	21.8122192	442.1898083	150.7902
*S*_8_	456.360696	467.863004	19.8943367	337.3776

**Table 3:** Case Study B: demand for 19000 megawatt, where *S*_1_ − *S*_8_ represent collection of sources operating on 8 fuel types.

	Firefly	BAT	GOA	IMVO
Price in PKR	327.227212	236.951036	233.3069629	**155.6167319**
Variable (MW)	19076.3946	19017.236	19023.02749	19044.11301
*S*_1_	6761.41122	4928.40531	5283.899334	3905.454
*S*_2_	2395.21169	2006.62552	1695.397916	1655
*S*_3_	1469.31172	204.258471	167.1485397	1674.898
*S*_4_	1701.02742	1102.9362	1720.041194	2433.113
*S*_5_	5583.69119	10116.7438	9981.020494	8160.88
*S*_6_	517.009725	153.65083	28.65642935	480.2648
*S*_7_	489.383474	93.3714342	137.4132769	394.7306
*S*_8_	159.348169	411.244455	9.450307236	339.7717

**Table 4:** Case Study C: demand for 23000 megawatt, where *S*_1_ − *S*_8_ represent collection of sources operating on 8 fuel types.

	Firefly	BAT	GOA	IMVO
Price in PKR	350.831986	263.667565	276.6948704	**187.660591**
Variable (MW)	23061.0476	23022.7254	23044.31656	23064.1461
*S*_1_	7331.80841	6292	7333.779792	5069.58882
*S*_2_	2493.78785	2618	1725.823048	1670.682
*S*_3_	1871.00046	219	56.0182213	2517.06661
*S*_4_	1756	1756	533.408886	2467.49605
*S*_5_	7685.67332	11612	11553.22811	9782.75386
*S*_6_	1104.62969	0.02326828	1089.543135	696.3726
*S*_7_	436.442721	525	462.5011979	448.716537
*S*_8_	381.705102	0.70208538	290.0141633	411.469586

**Table 5:** Electricity production of all 95 sources for five case studies.

	Case Study 1	Case Study 2	Case Study 3	Case Study 4	Case Study 5
Price in PKR	**1.12E + 08**	**1.51E + 08**	**1.84E + 08**	**1.97E + 08**	**2.16E + 08**
Variables (megawatts)	**15003.16**	**19003**	**23000.08**	**25000.3**	**27208.36**
TD (*x*_1_)	2379.137	1566.35	2938.164	3478	3476.16
MD (*x*_2_)	818.677	1000	905.7829	763.4957	983.48
GBHPP (*x*_3_)	516.5337	1450	1023.352	1347.306	1421.054
WD (*x*_4_)	130.7975	210.4076	177.7411	106.4834	181.5488
CB (*x*_5_)	38.74718	184	75.42442	174.4151	163.8171
DKD (*x*_6_)	42.56789	116.3349	116.0204	60.36495	127.4962
AKHPP (*x*_7_)	25.02909	0.415286	119.0034	33.87101	47.99439
KKHP (*x*_8_)	6.04886	15.92544	41.72064	72	19.27703
JagHP (*x*_9_)	14.17929	30	12.85605	8.998066	21.38508
JabHP (*x*_10_)	8.375935	10.57083	18.06	9.438497	0.704657
RG (*x*_11_)	6.866051	3.330527	0.380166	4.987555	9.745063
DHP (*x*_12_)	1.428187	13.53056	12.65834	20	2.044366
GHD (*x*_13_)	6.05225	0.280779	2.219217	9.40119	15.5921
NHP (*x*_14_)	6.647023	11.40453	7.595507	6.010873	13.86329
SHP (*x*_15_)	11.55404	11.2018	8.391618	11.16708	13.093
KHP (*x*_16_)	1.869716	1.137132	1.534735	2.692804	1.023365
RKHP (*x*_17_)	0.905093	1	1	0.651626	0.854247
CHPP (*x*_18_)	0.864139	0.815802	0.815802	0.965926	0.830172
GTPP (*x*_19_)	1305.331	1655	1655	1655	1645.54
MTPP (*x*_20_)	640.0394	546.27	1350	1350	1350
JTPP (*x*_21_)	149.5429	850	850	850	849.0554
FGTPP (*x*_22_)	148.9675	181.2005	188.0609	103.1949	217.891
MGTPP (*x*_23_)	4.385999	74.8375	155.4596	145.8149	168.1718
MGTPP (*x*_24_)	127.056	65.27735	166.15	34.1201	110.9528
KGTPP (*x*_25_)	112.8329	116.059	30.98832	0.567566	149.5035
LTPP (*x*_26_)	40.37149	0.883692	24.3518	120.5104	117.4427
FSPP (*x*_27_)	39.19613	54.27258	59	56.63659	55.09061
SGTPP (*x*_28_)	0.914076	21.86785	14.66417	22.23108	31.08445
PGTPP (*x*_29_)	27.30059	14.45997	0.589928	8.484009	23.07876
PTPP (*x*_30_)	12.70353	17	6.695689	16.85053	11.95603
KGTPS (*x*_31_)	91.79519	228.2708	247	127.6751	246.8065
KPC (*x*_32_)	91.12861	100	0.076503	16.21392	1.324039
GTPS (*x*_33_)	21.39859	30.86809	0.608317	41.91513	1.135833
TPS (*x*_34_)	1019.436	852.5889	921.7966	1132.252	1260
CCPP 1 (*x*_35_)	363.8285	375.9421	485.8476	538.1132	552.4153
CCPP 2 (*x*_36_)	67.16314	359.9164	551.1955	798.5884	899.1415
AES LL (*x*_37_)	12.48976	362	330.1038	341.4665	346.4409
AES PG (*x*_38_)	87.79272	289.7815	365	244.1401	364.9033
AEL (*x*_39_)	3.047575	29	29	12.08485	19.5939
AP (*x*_40_)	146.5024	194.7207	214.9626	225	225
AGL (*x*_41_)	153.9627	107.4664	152.7011	114.3378	113.5217
CHMC PL (*x*_42_)	75.13341	265.198	273.3111	255.6046	322.7874
DHA C L (*x*_43_)	68.73455	0.476099	6.545028	62.96757	70.30826
EPC (*x*_44_)	23.40477	98.656	37.10004	141.3346	152.0636
EPQL (*x*_45_)	130.7633	214.0389	222.3562	226.5	226.2002
FKPCL (*x*_46_)	95.2459	157	151.2643	27.8556	155.6898
FTSM (*x*_47_)	56.16674	104.4668	100.8249	76.57812	107.0448
FPCDL (*x*_48_)	10.1152	164.3581	127.2432	106.039	177.7063
GHLLP (*x*_49_)	101.702	56.26072	109.8266	140.2346	157.6599
GEPtvL (*x*_50_)	162.3922	188	144.4615	179.5249	131.9891
GEL (*x*_51_)	8.193736	89.19682	201.5	84.89962	201.5
GAEL (*x*_52_)	100.5592	92.35572	57.18626	110.4825	82.02294
HCPC (*x*_53_)	111.3358	58.26757	110.9772	43.47083	111.5484
HPGC (*x*_54_)	169.7313	0.807693	192.3602	125.0309	215.355
HUBCO NPP (*x*_55_)	147.8875	225	212.279	209.5134	223.5541
HUBCOHPP (*x*_56_)	148.0634	1292	1167.515	1292	1292
Iptvl (*x*_57_)	21.53738	158.6019	141.6202	164.2585	164.9125
JPG (*x*_58_)	84.13137	120	120	65.22738	119.424
KEL (*x*_59_)	43.50688	47.52342	103.8332	30.58684	130.8207
KAPCL (*x*_60_)	811.4451	1223.218	1185.71	1638	1638
LPL (*x*_61_)	153.0046	0.52402	94.67205	196.4468	182.2104
LPTL (*x*_62_)	124.1896	68.1917	50.95543	138.8238	198.6912
LEPCL (*x*_63_)	255.6012	589.7144	578.5459	660	658.6634
NCP (*x*_64_)	158.2538	200	95.85135	200	151.3611
NPL (*x*_65_)	32.48763	200	168.4331	168.8323	199.1059
OPCL (*x*_66_)	205.2865	0.89022	132.7586	65.89102	220.0356
REPGCL (*x*_67_)	30.20849	100.2075	52.99079	164	163.4477
RP (*x*_68_)	327.1605	276.0325	302.1523	412	411.8322
SPC (*x*_69_)	72.08175	75.02883	91.21424	109.1339	112.6283
SPPQ (*x*_70_)	101.0073	225	124.0716	211.05	221.962
SESL (*x*_71_)	208.1023	26.5598	66.00281	215.5041	200.3566
SSEL (*x*_72_)	220.5401	14.1361	321.1493	310.6587	343.2993
SNPCL (*x*_73_)	39.3573	44.14536	0.398791	20.11775	51.89066
SE (*x*_74_)	77.28166	11.07506	66.37944	23.84816	16.50386
SEPC (*x*_75_)	12.01473	71.11238	40.1338	47.43561	83.28035
SPGL (*x*_76_)	71.382	35.85756	10.86969	88.37954	113.884
TEL (*x*_77_)	110.2271	25.38428	113.5676	26.5028	94.01525
UchPL (*x*_78_)	735.2781	28.16507	990	990	989.7866
KANUPP (*x*_79_)	50.4151	92.10906	137	105.7683	134.7101
CHASNUPP-1 (*x*_80_)	282.9386	86.3647	275.8089	325	268.7866
CHASNUPP-2 (*x*_81_)	200.2746	243.2484	149.4219	325	320.8773
CHASNUPP-3 (*x*_82_)	191.8579	340	319.874	301.6324	214.0955
NPP (*x*_83_)	18.16426	173.693	374.8168	336.2965	420.7756
QezSP (*x*_84_)	49.71705	106.9033	70.03342	147.3463	126.7687
YEL (*x*_85_)	15.58682	2.334511	46.23074	24.48091	12.86986
MWPCL (*x*_86_)	41.01434	21.35616	27.94642	36.45242	45.21504
TGL (*x*_87_)	9.466357	25.20002	49	47.79046	47.90783
GAWPL (*x*_88_)	15.35919	18.91722	45.14516	15.24926	27.94244
MWEL (*x*_89_)	14.96827	21.99775	52	47.87871	46.00231
FFCEL (*x*_90_)	30.26861	42.68928	50	50	15.58795
ZEP (*x*_91_)	20.88768	14.37346	44.67045	30.45881	40.17728
TWEL (*x*_92_)	15.28468	12.1674	30	29.52479	28.65781
HCDPL (*x*_93_)	9.876255	20.00362	47.25205	31.9031	41.52962
FWEL 1 (*x*_94_)	43.69201	29.80095	45.98028	29.68556	22.61687
FWEPL 2 (*x*_95_)	6.404915	50	6.872093	21.65334	40.28912

**Table 6:** Top 20 electricity production sources according to the five case studies.

No. 1	No. 2	No. 3	No. 4	No. 5	No. 6	No. 7	No. 8	No. 9	No. 10	No. 11	No. 12	No. 13	No. 14	No. 15	No. 16	No. 17	No. 18	No. 19	No. 20
*x*_1_	*x*_19_	*x*_60_	*x*_3_	*x*_20_	*x*_56_	*x*_34_	*x*_78_	*x*_21_	*x*_2_	*x*_36_	*x*_63_	*x*_35_	*x*_68_	*x*_83_	*x*_37_	*x*_38_	*x*_72_	*x*_74_	*x*_81_

**Table 7:** Per megawatt cost of electricity for different fuel types.

Fuel type	Price in PKR per megawatt
Hydel	2500
LNG	9070
Furnace oil	11050
High-speed diesel	17960
Coal	12080
Solar	16950
Wind	16630
Nuclear	6860
Regasified liquefied natural gas	11270

## Data Availability

The data used to support the findings of this study are available from the corresponding author upon request.

## Nomenclature

TD: Tarbela Dam (*x*_1_)
MD: Mangla Dam (*x*_2_)
GBHPP: Ghazi Barota Hydropower Project (*x*_3_)
WD: Warsak Dam (*x*_4_)
CB: Chashma Barrage (*x*_5_)
DKD: Duber Khwar Dam (*x*_6_)
AKPH: Allai Khwar Hydropower Plant (*x*_7_)
KKPL: Khan Khwar Hydropower Plant (*x*_8_)
JHP: Jagran Hydropower Plant (*x*_9_)
JabHP: Jabban Hydropower Plant (*x*_10_)
RB: Rasal Barrage (*x*_11_)
DHP: Dargai Power Plant (*x*_12_)
GZD: Gomal Zam Dam (*x*_13_)
NHP: Nandipur Hydropower Plant (*x*_14_)
SHP: Shadiwal Hydropower Plant (*x*_15_)
KHP: Khuram Hydropower Plant (*x*_16_)
RKHP: Renela Khuard Hydropower Plant (*x*_17_)
CHPP: Chatral Hydropower Plant (*x*_18_)
GTPP: Guduu Thermal Power Plant (*x*_19_)
MTPP: Muzaffargarh Thermal Power Plant (*x*_20_)
JTPP: Jamshoro Thermal Power Plant (*x*_21_)
FGTPP: Faisalabad Gas Turbine Power Plant (*x*_22_)
MGTPP: Multan Gas Turbine Power Plant (*x*_23_)
KGTPP: Kotri Gas Turbine Power Plant (*x*_24_)
LTPP: Larkana Thermal Power Plant (*x*_25_)
FSPP: Faisalabad Steam Power Plant (*x*_26_)
SGTPP: Shahdra Gas Turbine Power Plant (*x*_27_)
PGTPP: Panjgur Gas Turbine Power Plant (*x*_28_)
QTPP: Quetta Thermal Power Plant (*x*_29_)
PTPP: Pasni Thermal Power Plant (*x*_30_)
KPC: Korangi Power Complex (*x*_31_)
KGTPS: Korang Gas Turbine Power Plant Station (*x*_32_)
GTPS: Gas Turbine Power Station (*x*_33_)
KPS: Thermal Power Plant (*x*_34_)
CCPP1: Combined Cycle Power Plant 1 (*x*_35_)
CCPP2: Combined Cycle Power Plant 2 (*x*_36_)
AESLL: AES Lalpir Limited (*x*_37_)
AESPG: AES Pak Gen (*x*_38_)
AEL: Altern Energy Limited (*x*_39_)
AP: Altes Power (*x*_40_)
AGL: Attock Gen Limited (*x*_41_)
CMECP: CMEC Power Pvt. (*x*_42_)
DHACL: DHA Cogen Limited (*x*_43_)
EPC: Eastern Power Company (*x*_44_)
EPQL: Engro Powergen Qaidpur Limited (*x*_45_)
FKPP: Fauji Kabirwala Power Plant (*x*_46_)
FTSM: First-Star Modaraba (*x*_47_)
FPCDL: Foundation power company Daharki Limited (*x*_48_)
GHLLP: Grang Holding Limited Power Plant (*x*_49_)
GEPtvl: Green Electric Pvt. Limited (*x*_50_)
GEL: Gujranwala Energy Limited (*x*_51_)
DAEL: Gul Ahmad Energy Limited (*x*_52_)
HCPC: Habibullah Coastal Power Company (*x*_53_)
HPGC: Halmore Power Generation Company (*x*_54_)
HUBCONPP: HUBCO Narowal Power Plant (*x*_55_)
HUBCOHPP: HUBCO Hub Power Plant (*x*_56_)
IPtvL: Intergen PTV Limited (*x*_57_)
JPG: Japan Power Plant (*x*_58_)
KEL: Kohinor Energy Limited (*x*_59_)
KAPCL: Kot Addu Power Company Limited (*x*_60_)
LPL: Liberty Power Limited (*x*_61_)
LPTL: Liberty Power Tech. Limited (*x*_62_)
LEPCL: Lucky Electric Power Company Limited (*x*_63_)
NCP: Nishat Chunian Power (*x*_64_)
NPL: Nishat Power Limited (*x*_65_)
OPCPrvL: Orient Power Company (Pvt.) Limited (*x*_66_)
REPGC: Radian Energy Power Generation Company (*x*_67_)
RPK: Rousch Power, Khanewal (*x*_68_)
SPC: Saba Power Company (*x*_69_)
SPPQ: Saif Power Plant, Qadirabad (*x*_70_)
SECL: Sapphire Electric Company Limited (*x*_71_)
SSEL: Siddiqsons Energy Limited (*x*_72_)
SNPCL: Sindh Nooriabad Power Company (Pvt.) Limited (*x*_73_)
SE: Sitara Energy (*x*_74_)
SEPC: Southern Electric Power Company Limited (*x*_75_)
SPGL: Star Power Generation Limited (*x*_76_)
TEL: Tapal Energy Limited (*x*_77_)
UchPL: Uch (Uch-I and Uch-II) Power Limited (*x*_78_)
KANUPP: KANUPP (*x*_79_)
CHASNUPP-1: CHASNUPP-1 (*x*_80_)
CHASNUPP-2: CHASNUPP-2 (*x*_81_)
CHASNUPP-3: CHASNUPP-3 (*x*_82_)
NPP: Nandipur Power Project (*x*_83_)
QeaSP: Quaid-e-Azam Solar Park (*x*_84_)
YEL: Yunus Energy Limited (*x*_85_)
MWPCL: Metro Wind Power Co Limited (*x*_86_)
TGL: Tenaga Generai Limited (*x*_87_)
GAWPL: Gul Ahmed Wind Power Limited (*x*_88_)
MWEL: Master Wind Energy Limited (*x*_89_)
FFCEL: FFC Energy Limited, Jhimpir (*x*_90_)
ZEPJ: Zorlu Enerji Pakistan, Jhimpir (*x*_91_)
TWEL: Tapal Wind Energy Limited (*x*_92_)
HCDPL: HydroChina Dawood Power Limited (*x*_93_)
FEW-1L: Foundation Wind Energy-I Limited (*x*_94_)
FEW-2PT: Foundation Wind Energy-II Private Limited (*x*_95_).
